# Supplementary material for: Learning curve and surgical outcome of robotic assisted colorectal surgery with ERAS program
Source: Sci Rep. 2022 Nov 29;12:20566. doi: 10.1038/s41598-022-24665-w (PMC9709162; doi:10.1038/s41598-022-24665-w)
Supplement: Supplementary file 1 — Supplementary Information 1. [file 41598_2022_24665_MOESM1_ESM.zip › LSC Raw data-Ñ[▒K/058/OP N_ñw╝╨░O▒Kñσ.pdf]

請選擇...reporter

臺中榮民總醫院
手術室護理記錄

[redacted]

基本資料

- 室別： 二醫手術室-38室，手術科別： CRS，手術類別： 常規，日期： 2019/12/17
- 進入時間： 1125 ； 麻醉開始時間： 1150 ； Time Out時間： 1222 ； 劃刀時間： 1225 ； 手術結束時間： 1645 送出時間： 1700 ；
- 麻醉方式： GE
  診斷：rectum tumor bleeding.
  手術名稱：1.Low anterior resection with colo-anal anastomosis2.Ileostomy3.Laparoscopy4.
  術前預防性抗生素：
  - 抗生素名稱 ： Veterin 1GM IVD
  - 時間： 1215
  - 上次簽章: 楊智雯
  ，追加預防性抗生素：
- 手術醫師： 林俊余，林昌霖，陳瑢夫
- 麻醉醫師： VS 施涵薰
-
  - 刷手護士A ： 李宜芳，時間： 1125-1200，刷手護士B ： 許育庭，時間： 1125-1200，接替刷手一： 王心俞，時間： 1200-1300，接替刷手二： 許育庭，時間： 1230-1530
  - 流動護士A ： 楊智雯，時間： 1125-1230，接替流動一： 李宜芳，時間： 1230-1700
- 更多接替及流動護士： 接替刷手三： 楊智雯，時間： 1300-1530，接替刷手四： 王毓均，時間： 1530-1700

皮膚狀況

A-V Shunt： 無
術前： 不完整
部位(術前)：

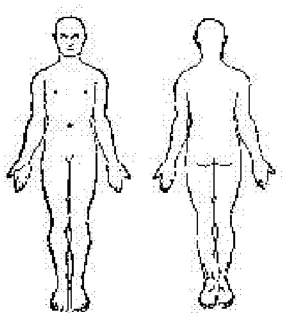

全身多處黑斑

記錄者： 上次簽章: 李宜芳
術後： 不完整
部位(術後)：

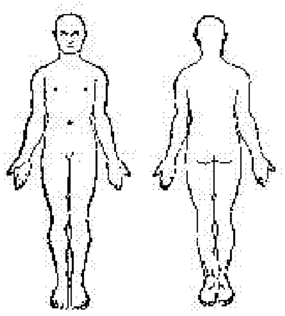

同術前

記錄者： 上次簽章: 李宜芳

手術準備

手術臥式 單一手術臥式

-
  - 手術臥式： 截石臥式
  - 使用附件： 溫毯 38 ℃，頭圈，腳架
  - 皮膚準備： 10%Aq Betadine，Hibiscrub，其它： 2% CHOLRHEXIDINE
  - 電燒： 使用電燒: 電極片位置： 右大腿，記錄者： 上次簽章: 李宜芳

止血帶T： 無

植入物

- 植入物名稱/號碼/數量/部位： EndoGIA 60mm-4.1mm釘

, 記錄者： 上次簽章: 李宜芳

標本送檢

- 細菌培養 無
- 細胞學 無
- 冰凍切片 無
- 病理標本
  組織名稱一： distal cut end
  ,
  組織名稱二： RS colon
- 截肢 無

標本交付 標本： 2 袋，標本： 0 盒，標本： 0 瓶，細胞學： 0 管，Culture Tube： 0 管，被交付醫師： CR 林昌霖，

- 記錄者： 上次簽章: 李宜芳
- 時間： 1615

傷口術後敷料

傷口敷料： 傷口敷料： 無

- 請於圖上標示部位：

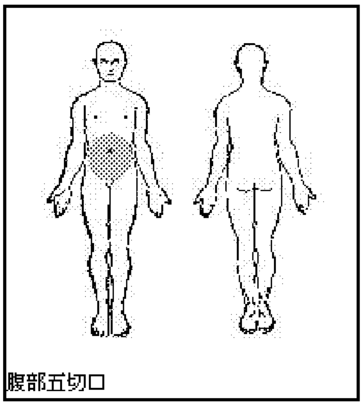

- 傷口敷料： MEPORE

導管

導管	天數	導管資訊	插入日期	插入時間	更換日期	拔除日期	拔除時間	拔除原因	預計拔除/更換	預計拔除/更換日期
Foley(1)	1	號碼:16 Fr.; 部位:膀胱;	2019/12/17	1220						
Rod,Stoma Supporting (1)	1	號碼:壹個 ; 部位:左腹;	2019/12/17	1620						
Penrose Drain(1)	1	號碼:12 mm.; 部位:骨盆腔;	2019/12/17	1610						
Sump NG Tube(1)	1	號碼:16 Fr.; 深度:55CM; 部位:胃;	2019/12/17	1150						

用物計數

- 手術中出血量： 100 c.c.
- 手術中尿量： 300 c.c.
-
  - 其它： 無

用物計數： 多切口同時縫合

- 1.器械： 正確，2.縫針： 正確，3.刀片： 正確，4.紗布： 正確，5.腹部墊： 未使用，6.紗球： 未使用，7.PEANUTS ： 未使用，8.紗條： 未使用，9.綵帶： 未使用，10.針頭： 未使用，11.Vessel loop： 未使用，12.Boots： 未使用，13.Jelco： 未使用，14.棉條： 未使用，
  - 第一次：
    - 刷手護士 ： 王毓均
    - 流動護士： 上次簽章: 李宜芳
    - 時間： 1615

  ,
  - 第二次：
    - 刷手護士 ： 王毓均
    - 流動護士： 上次簽章: 李宜芳
    - 時間： 1620

  ,
  - 第三次：
    - 刷手護士 ： 王毓均
    - 流動護士： 上次簽章: 李宜芳
    - 時間： 1625

備註

備註：NIL

- 記錄者： 上次簽章: 李宜芳
- 時間： 1615

填寫護理人員 李宜芳

正式摘要

請選擇...reporter

doctorRank:B
